# Inter-species and inter-colony differences in elemental concentrations in eggshells of sympatrically nesting great cormorants *Phalacrocorax carbo* and grey herons *Ardea cinerea*

**DOI:** 10.1007/s11356-018-3765-5

**Published:** 2018-11-27

**Authors:** Dariusz Jakubas, Ignacy Kitowski, Dariusz Wiącek, Szymon Bzoma

**Affiliations:** 1 Department of Vertebrate Ecology and Zoology, University of Gdańsk, Wita Stwosza 59, PL-80-308 Gdańsk, Poland; 2 State School of Higher Education in Chełm, Pocztowa 54, PL-22-100 Chełm, Poland; 3 Institute of Agrophysics, Polish Academy of Sciences, Doświadczalna 4, PL-20-290 Lublin, Poland; 4 Grupa Badawcza Ptaków Wodnych KULING, Świerkowa 34/7, PL-81-526 Gdynia, Poland

**Keywords:** Contamination, Environmental pollution, Piscivores, Waterbirds

## Abstract

We compared the concentrations of 17 heavy metals and essential elements in post-hatching eggshells of two waterbirds, the obligate piscivorous great cormorant *Phalacrocorax carbo* (GCM) and the more omnivorous grey heron *Ardea cinerea* (GHR), breeding sympatrically in eight mixed colonies in Poland. We found significant inter-species and inter-colony differences in the levels of most of the elements. GHR had significantly higher concentrations of Al, which can be explained by its very low stomach pH: an acidic environment favours the release of Al compounds. Differences in Mn, Ni, Cu, Se and Hg concentrations can be attributed to the various contributions of fish and other aquatic organisms to the diet, and to the exploration of different habitats (GCM exclusively aquatic, GHR a wider range) and microhabitats (GCM, in contrast to wading GHR, dive for food, exploring the whole depth range of water bodies), differently exposed to contamination by those elements from sediments. Inter-colony differences were related to the level of industrialisation. We recorded higher levels of some elements in the eggshells (Fe, Mn in both species and Cr, Ni and Zn in GCM) collected in industrialised areas, which may be associated with the negative environmental impact of industrial areas.

**Electronic supplementary material:**

The online version of this article (10.1007/s11356-018-3765-5) contains supplementary material, which is available to authorized users.

## Introduction

Waterbirds like cormorants and herons are top predators: as such, they are exposed to a variety of contaminants, which they are liable to bioaccumulate. Many studies have indicated that this group of birds could be a good biomonitor of heavy metal pollution of the environment and local contamination around breeding sites (e.g. Becker 2003; Malik and Zeb 2009; Zhang and Ma 2011). Gregariously nesting waterbirds provide an opportunity to collect various samples, including eggs, from many individuals and species at once. Avian post-hatching eggshells are commonly used in bio-indication and environmental monitoring studies (Khademi et al. 2015; Simonetti et al. 2015; Kitowski et al. 2017), as they can be collected non-invasively in breeding colonies. During egg formation, some contaminants are removed from the female body and are sequestered in the eggs, including the shells (Orlowski et al. 2014; Orlowski et al. 2016; Luo et al. 2016). Some metals, like Hg, Pb and Al, may impair eggshell structure (Nyholm 1981; Scheuhammer 1987; Eeva and Lehikoinen 1995; Lucia et al. 2010), thereby affecting hatchability. The shells and contents of eggs may differ in levels of particular elements, e.g. Cd, Pb and Mn (Agusa et al. 2005; Hashmi et al. 2013; Kim and Oh 2014); However, level of some elements in egg content and eggshell may be strongly correlated indicating that, e.g. eggshell concentrations of Cd, Pb and Cu can mirror their levels in the egg contents (Kim and Oh 2014). Biomonitoring studies can take advantage of this. A study of Zn and Cu concentrations in tit eggs revealed no differences in egg contents but marked differences in eggshells between populations breeding in polluted and unpolluted areas (Dauwe et al. 1999). This result indicates that the levels of both elements in the egg contents are homeostatically controlled and that the contents are a less suitable bio-indicator than the shell. A signal from eggshells represents relatively short period of time, i.e. pre-laying (Becker 2003) and various areas depending on the strategy of acquiring nutrients for egg production. In the case of capital breeders, nutrients are stored before breeding, e.g. at stopover sites during spring migration. In contrast, income breeders acquire nutrients locally during the pre-laying period (Stephens et al. 2009).

In this study, we investigated the concentrations of heavy metals and other elements in post-hatched eggshells of two waterbirds—great cormorant *Phalacrocorax carbo* (GCM) and grey heron *Ardea cinerea* (GHR). Despite their ostensible similarity, these species differ in their dietary composition and foraging techniques. Whereas the GCM diet consists entirely of fish (Cramp 1998), GHR is a highly opportunistic predator, with a diet varying widely according to habitat and season; depending on location, it may be dominated by fish, crustaceans or mammals (Cramp 1998; Kushlan and Hancock 2005). GHR may respond to changes in prey availability by switching to other available prey (e.g. Jakubas and Manikowska 2011). Their feeding techniques also differ: GCM mainly dives for fish, whereas GHR catches its prey by grabbing or stabbing, rarely by surface swimming or aerial plunging. Moreover, the anatomy of GHR restricts it to the shallow water zone of aquatic habitats (Cramp 1998).

Since GCM and GHR both adopt the income breeder strategy for acquiring nutrients for egg production (Hobson 2006; Cotin et al. 2012), their maternal investments (including eggshells) should correspond to the contamination of the local breeding environment (Stephens et al. 2009). Being top predators in freshwater ecosystems, both species are considered important ‘indicator species’ in environmental monitoring, as they can bio-magnify and bio-accumulate toxic and essential elements, pesticides and various pollutants (e.g. Cooke et al. 1982; Scharenberg 1989; Newton et al. 1993). Both species acquire nutrients for egg production locally, so we expected that the element levels accumulated in their eggs would reflect local contamination of their food and environment.

In this study, we aimed to:

1. Measure the concentrations of selected elements and then compare them between the two species,
2. Investigate whether the concentrations of elements, including heavy metals, in the eggshells of GCM and GHR varied among the colonies studied;
3. Determine whether the concentrations of elements are related to the level of industrialisation in the vicinity of the breeding colonies.
4. Determine possible common sources of elements in the two species.

Given the interspecific differences in diet (GCM—an obligate piscivore and GHR—an opportunistic, facultative piscivorous predator; Cramp 1998), we expected some variations in elemental concentrations, e.g. more Cu, Mn, Se and Hg in GCM as a fish-rich diet favours the accumulation of these elements in eggs (Monteiro and Furness 1995; Grajewska et al. 2015; Ackerman et al. 2016).

In view of the habitat differences in the vicinity of the breeding colonies, we expected some inter-site differences in elemental concentrations. Some of the colonies studied are situated close to highly industrialised, urbanised and densely populated areas with smelting plants, coal mines, oil refineries, e.g. the Upper Silesian Industrial Region and the Warsaw conurbation (the capital of Poland, 1.75 million inhabitants). Both GCM and GHR forage in the potentially contaminated aquatic habitats of such areas, where long-term emissions of Pb, Cd and Ni from industry, the energy sector and transport are usual (Majewski and Lykowski 2008; Rogula-Kozlowska et al. 2013; Werner et al. 2014; Holnicki et al. 2016), and the levels of these elements in watercourses and their sediments are elevated (Gierszewski 2008; Rzetala et al. 2013; Rzetala 2016; Barbusinski et al. 2012; Dmochowski and Dmochowska 2011). We therefore anticipated that Pb, Cd and Ni levels in eggshells from colonies situated in highly industrialised areas would be higher than those from colonies situated in areas with less industry.

## Materials and methods

### Material collection

**Fig. 1:**
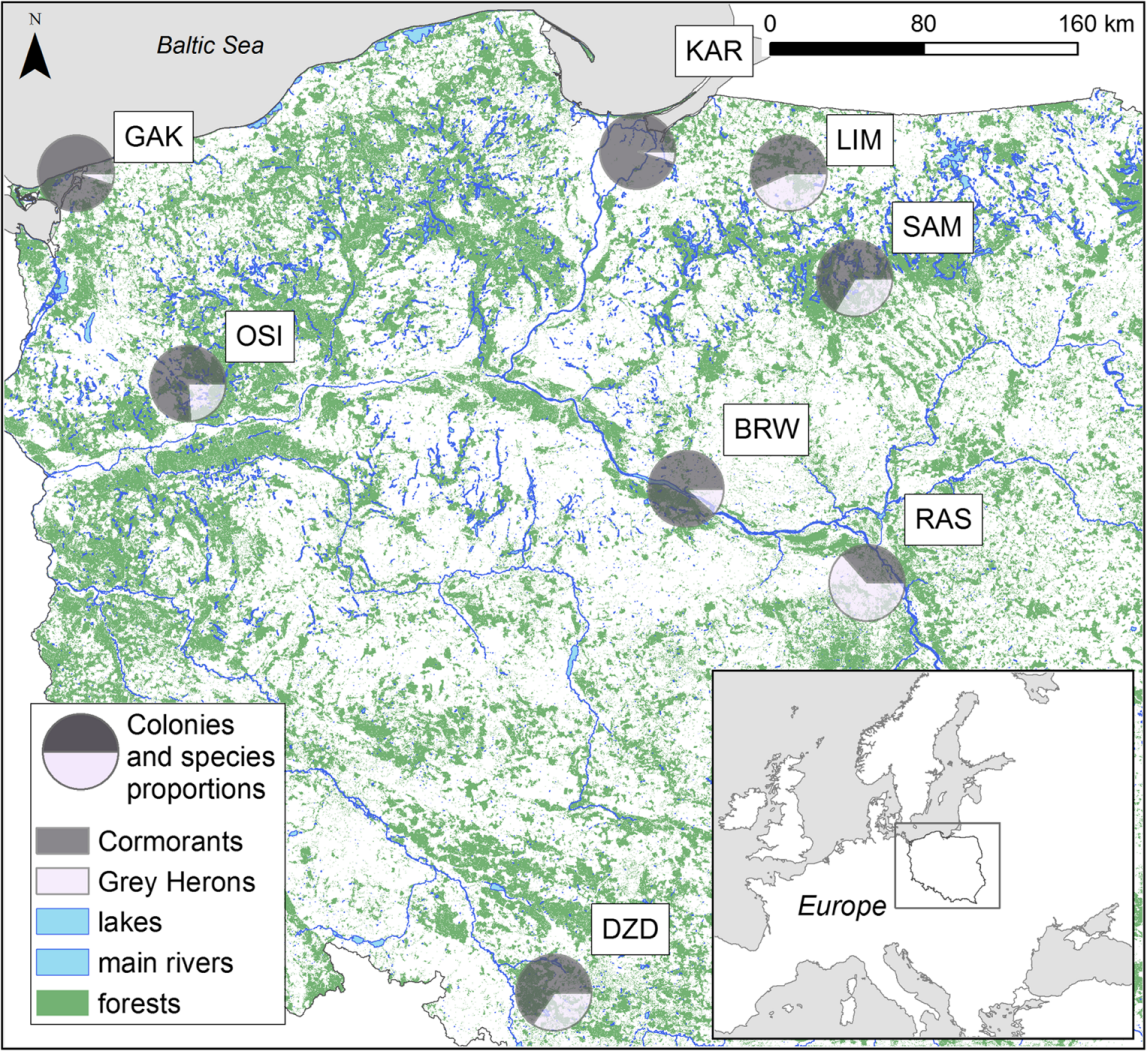
Study area showing the positions all the mixed colonies of great cormorants and grey herons studied here (the pie charts show the proportions of nests in particular colonies). The size of the pie charts reflects the 20-km buffers around the colonies—potential foraging areas. For the colony codes—see Table 1

We collected post-hatched eggshells during short visits to eight mixed colonies in Poland (Fig. 1, Table 1) during the chick-rearing period in April–June 2015. During the visits, all occupied nests of both species were counted by Szymon Bzoma. Eggshells were picked up at random from the ground beneath the nesting trees, packed in plastic bags and transported to the laboratory.

**Table 1:** Characteristics of the mixed colonies of great cormorant (GCM) and grey heron (GHR). Colony location—see Fig. 1

Colony name and code	No. of breeding pairs*	Characteristics of the colony**
Lake Osiek OSI	GCM: 290 GHR: 91	The largest lake in the Dobiegniew Lake District (area 5.39 km^2^, mean depth 9.3 m) situated in a wooded area. The lake is subject to minor tourist pressure. Birds forage in numerous lakes in this Lake District.
Lake Limajno LIM	GCM: 94 GHR: 72	Colony on an island in the lake. Lake Limajno (area 2.32 km^2^, mean depth 9 m) is situated in a wooded area and is subject to very little tourist pressure. Birds forage in neighbouring lakes in the Masurian Lake District.
Sasek Mały SAM	GCM: 155 GHR: 79	A small (area 3.2 km^2^, mean depth 1.6 m) kettle lake in a wooded area. Tourist pressure here is minimal. Birds forage in numerous lakes in the Masurian Lake District
Raszyn RAS	GCM: 19 GHR: 33	Colony in a carp pond complex (area 1.1 km^2^, mean depth 1 m). The birds forage in ponds and the River Vistula. It is situated in a highly urbanised area, very close to Warsaw, the biggest city in Poland, with ironworks, power plants, factories and heavy traffic.
Gardzka Kępa GAK	GCM: 2735 GHR: 146	Colony situated on an island in the strait between the Szczecin Lagoon and the Pomeranian Bay (Baltic Sea). The Szczecin Lagoon is a large (687 km^2^, mean depth 3.8 m) lagoon in the Odra River estuary. Birds forage at sea, in the lagoon and on the lakes.
Brwilno BRW	GCM: 2372 GHR: 320	Colony situated on an island in the Włocławek Reservoir. This is a large reservoir (70.4 km^2^) that formed when the Vistula was dammed: mean depth 5.5 m. It is situated close to a large oil refinery. Birds forage in the Włocławek Reservoir and adjacent lakes.
Kąty Rybackie KAR	GCM: 5300 GHR: 279	Colony situated on the Vistula Spit on the coast of the Gulf of Gdańsk (Baltic Sea). Birds forage at sea and in the lagoon (both species) and adjacent meadows (grey heron). The Vistula Lagoon is a large (838 km^2^) brackish water lagoon in the Vistula river delta: mean depth 2.7 m.
Dzierżno Duże DZD	GCM: 256 GHR: 134	The colony is situated close to the Dzierżno Duże reservoir (area 6.15 km^2^, max depth 20 m), formed in flooded mineral workings and acting as a sedimentation basin for the contaminated River Kłodnica carrying polluted waters from the Upper Silesian Industrial Region. The vicinity of the colony is highly urbanised and industrialised; the level of environmental pollution from coalmines, ironworks and coking plants is also high. Birds forage in the reservoir and adjacent rivers.

*Estimated by Szymon Bzoma

**After Robakiewicz (1993), Choinski (2006), Gierszewski (2008), Barbusinski and Nocon (2011), Walczuk and Romanowski (2013), Rzetala (2016)

We collected a total of 94 GCM eggshells (12 at Brwilno (BRW), 12 at Dzierżno Duże (DZD), 12 at Gardzka Kępa (GAK), 12 at Kąty Rybackie (KAR), 12 at Limajno (LIM), 12 at Osiek (OSI), 10 at Raszyn (RAS) and 12 at Sasek Mały (SAM)] and 98 GHR eggshells (12 at BRW, 13 at DZD, 12 at GAK, 15 at KAR, 12 at LIM, 10 at OSI, 12 at RAS and 12 at SAM)). The characteristics of all colonies are listed in Table 1.

### Laboratory analyses

Before taking any measurements, we removed the inner membrane from the eggshells, then washed these with deionised water, rinsed them with acetone and ground them in a ceramic mortar. We divided each eggshell into two sub-samples and analysed them in duplicate. We used the mean value per eggshell in all calculations.

We initiated mineralisation by pouring 10 mL of concentrated HNO_3_ (Sigma-Aldrich, Poland) over 500 ± 1 mg of eggshell and wet washing the sample. The process then took place in the following steps:

1. 15 min from room temperature to 140 °C,
2. 5 min at 140 °C,
3. 5 min from 140 °C to 170 °C,
4. 15 min at 170 °C,
5. Cooling to room temperature (various).

The pressure during mineralisation did not exceed 12 bars.

To determine the concentrations of the particular elements, we used an iCAP 6500 Series inductively coupled plasma optical emission spectrometer from Thermo Scientific (USA), equipped with a charge injection device. We used the following instrumental settings (iCAP 6000 Series Hardware Manual, 2010): RF generator power = 1150 W, RF generator frequency = 27.12 MHz, coolant gas flow rate = 16 L min^−1^, carrier gas flow rate = 0.65 L min^−1^, auxiliary gas flow rate = 0.4 L min^−1^, max integration time = 15 s, pump rate = 50 rpm, axial viewing configuration, three replicates and flush time = 20 s.

We used the following multi-element stock solutions as standards (Inorganic Ventures, Inc.):

1. Analityk—46: Cu, Fe, Mg, in 5% HNO_3_—1000 μg mL^−1^,
2. Analityk—47: Al, As, Cd, Cr, Pb, Mn, Hg, Ni, Se, Sr, V, Zn in 10% HNO_3_—100 μg mL^−1^,
3. Analityk—83: Ca, K, Mg, in 2% HNO_3_—1000 mg L^−1^,
4. CGMO1-1: Mo in H_2_O with traces of NH_4_OH—1000 μg mL^−1^.

To validate the analytical method, we calculated the following parameters (Table S1):

1. Linearity: the ability of the method to obtain test results proportional to the concentration of the analyte, expressed by the Pearson correlation coefficient.
2. Limit of detection: the lowest quantity of a substance that can be distinguished from the absence of that substance.
3. Recovery percentage: after random selection of three samples and supplying them individually with known amounts of the analytical standard, we calculated the mean percentage recoveries of the target elements using the equation:

Recovery [%] = (*C*_E_/*C*_S_ × 100),

where *C*_E_ is the experimental concentration determined from the calibration curve and *C*_S_ is the spiked concentration.

### Statistical analyses

To investigate element concentration patterns in post-hatched eggshells, we performed a principal component analysis (PCA) to reduce the number of variables to a few new ones called factors, representing groups of elements with significantly correlated concentrations. Since the concentrations of all elements were measured in the same units (mg kg^−1^ dw), we did the PCA on a variance–covariance matrix.

To find groups of elements with high degrees of association/correlation, we carried out a hierarchical cluster analysis (HACA) to find groups of elements and elements with high degrees of association. We did this using Bray–Curtis similarity, pairing the group method as the linkage method; for each cluster obtained, we calculated the bootstrap probability (BP) using multiscale bootstrap resampling. BP of a cluster can take any value between 0 and 100—this indicates how well the data supports the cluster (Hammer et al. 2001). Only clusters with BP ≥ 95 were taken into consideration. To determine how well the generated clusters represent dissimilarities between objects, we calculated the cophenetic correlation coefficient with values close to 0 indicating poor clustering, and close to 1, indicating good clustering. A high degree of association (e.g. clustering in one group) of element concentrations can be used to identify common sources of contaminants (Hashmi et al. 2013).

To investigate inter-group differences in element concentrations, we used the following methods:

1. Multivariate (for all elements together):

- Multivariate two-way PERMANOVA (non-parametric MANOVA based on the Bray–Curtis measure; Anderson 2001) with fixed factors (species and colony) and their interaction as explanatory variables; to further study effects with more than two levels (i.e. colony and species × colony interaction), identified as statistically significant by two-way PERMANOVA, we used one-way PERMANOVA as a post hoc test;
- The similarity percentage breakdown (SIMPER) procedure to assess the average percentage contribution of individual factors to the dissimilarity between objects in a Bray–Curtis dissimilarity matrix (Clarke 1993).

2. Univariate analysis (for particular elements) using univariate PERMANOVA (non-parametric MANOVA based on the Bray–Curtis measure; Anderson 2001) with fixed factors (species and colony) and their interaction as explanatory variables; to further study the effects of species and species × colony interaction, we performed one-way PERMANOVA as a post hoc test. We did not test the colony effect with respect to differences in foraging strategies and diet composition; we expected species-specific effects.

We performed all the analyses on log (*x* + 1) transformed data.

To assess the influence of industrial pollution on concentrations of particular elements in post-hatching eggshells collected in the colonies, we compared levels of elements in eggshells from colonies situated in industrialised and non-industrialised areas using the Mann-Whitney *U* test separately for GCM and GHR. The colonies located in industrialised areas were those at BRW (close to an oil refinery), DZD (near a fertiliser plant and an ironworks) and RAS (close to Warsaw, the largest city in Poland, with power plants, ironworks and heavy traffic etc.) (Table 1). The remaining colonies were considered as being situated in non-industrialised areas.

We conducted the statistical analyses using STATISTICA 12.0 (StatSoft Inc. 2014) and PAST 3.0 (Hammer et al. 2001).

## Results

### Variation in composition of trace elements in post-hatched eggshells

**Fig. 2:**
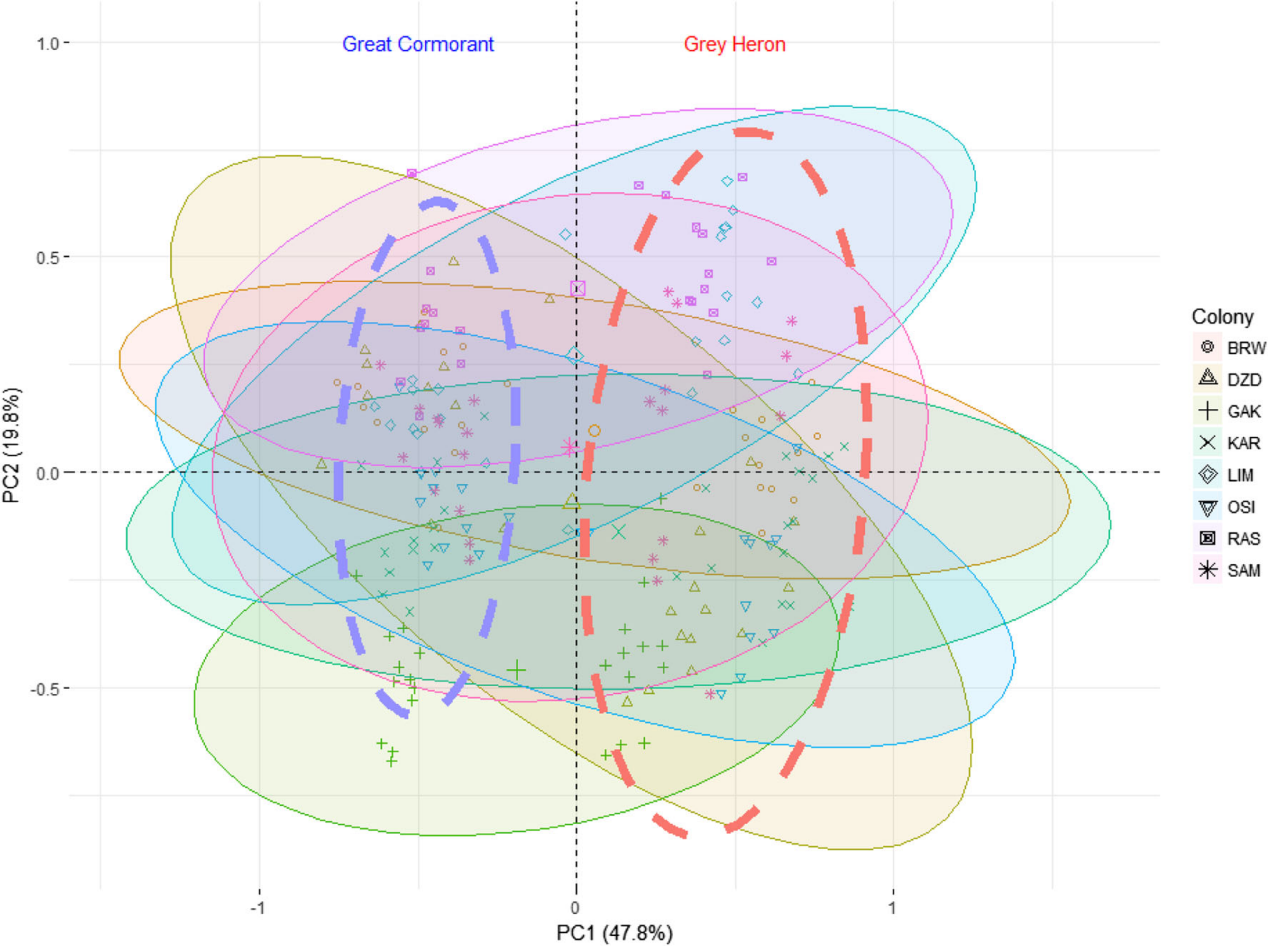
PCA biplot showing elemental concentrations in eggshells of great cormorants and grey herons breeding in eight mixed colonies in Poland. The filled convex hulls contain samples from one colony. The dashed convex hulls represent species. Colony codes—see Table 1

Principal component analysis (PCA) revealed that 82.4% of the total variance in the elemental concentrations of the eggshells was explained by the three axes (Table 2). PC1 explained 48% of the total variance and was highly positively correlated with the concentration of Al (*r* = 0.76) (Table 2). PC2 explained 20% of the total variance and was highly positively correlated with the Zn level (*r* = 0.75) (Fig. 2). PC3 explained 15% of the total variance and was moderately negatively correlated with the Sr concentration (*r* = 0.60) (Table 2). Both species clustered on opposite sides of the PC1 axis in the PCA plot (Fig. 2). Some colonies clustered in similar positions in relation to the PC2 axis in both species (e.g. KAR-DZD; LIM-RAS) (Fig. 2).

**Table 2:** The principal component loadings for the elements (log (*x* + 1) transformed) in great cormorants and grey herons; strongly and highly correlated values (*r* > |0.70|) are shown in italics

Element	Axis 1	Axis 2	Axis 3
Al	*0.762*	− 0.037	0.228
As	0.110	0.078	− 0.092
Ca	0.089	− 0.001	− 0.002
Cd	− 0.001	0.004	− 0.005
Cr	0.037	0.012	0.070
Cu	0.171	− 0.016	0.105
Fe	0.218	0.288	0.537
Hg	0.013	0.042	− 0.016
Mg	0.120	− 0.035	0.051
Mn	− 0.308	0.396	0.466
Mo	− 0.009	− 0.003	0.011
Ni	− 0.438	0.000	0.194
Pb	0.035	− 0.003	0.058
Se	0.050	0.144	− 0.081
Sr	− 0.123	− 0.412	0.601
V	0.008	0.007	− 0.024
Zn	0.036	*0.748*	− 0.081
Eigenvalues	0.246	0.102	0.076
Total variance explained (%)	47.8	19.9	14.8

**Fig. 3:**
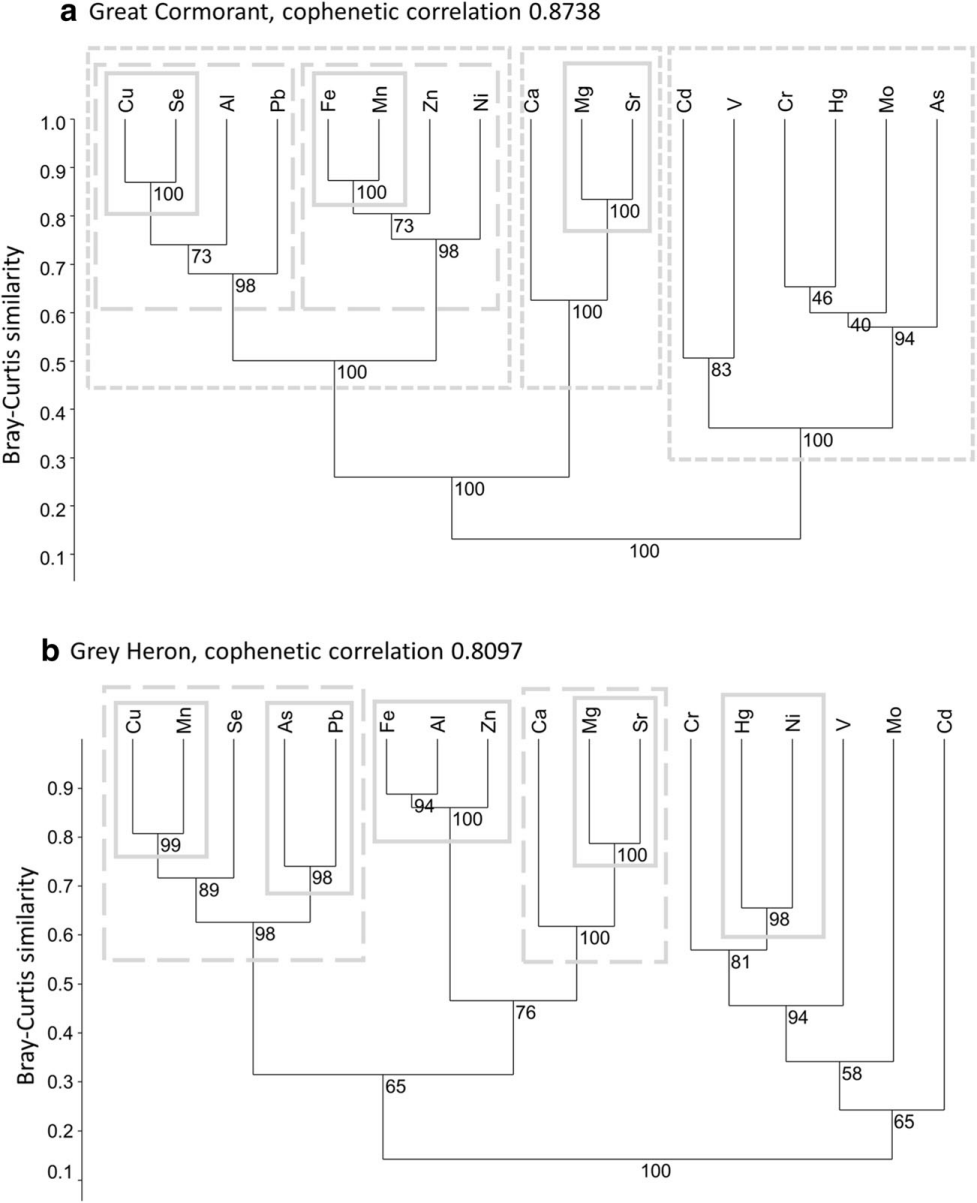
Hierarchical dendrogram of the target elements in post-hatched eggshells of great cormorants (**a**) and grey herons (**b**) obtained using the paired group method and Bray–Curtis similarity. The numbers below the branches indicate bootstrap probabilities (bootstrap *n* = 1000)

The hierarchical cluster analysis (HACA) for GCM (cophenetic correlation 0.8738) identified three main significant clusters grouping elements: Cd–V–Cr–Hg–Mo–As, Ca–Mg–Sr and Cu–Se–Al–Pb–Fe–Mn–Zn–Ni (Fig. 3a). We distinguished the subgroup Mg–Sr (BP = 100) in the second group, and two subgroups—Fe–Mn–Zn–Ni (BP = 100, with the distinctive inner subgroup Fe–Mn) and Cu–Se–Al–Pb (BP = 100, with the distinctive inner subgroup Cu–Se)(Fig. 3a)—in the third group.

HACA for GHR (cophenetic correlation 0.8097) identified some significant clusters grouping elements: Cu–Mn–Se–As–Pb (BP = 98), Fe–Al–Zn (BP = 100), Ca–Mg–Sr (BP = 100) and Hg–Ni (BP = 100) (Fig. 3b). In the first group, we distinguished two subgroups with high BP (≥ 95) values—Cu–Mn (BP = 99) and As–Pb (BP = 98). In the second group, we found only one significant subgroup—Mg–Sr (BP = 100).

### Factors affecting concentrations of all elements combined

We found that the concentrations of all the elements combined were significantly affected by species (multivariate two-way PERMANOVA, similarity measure: Bray–Curtis, *F*_2,176_ = 379.4, *p* = 0.0001), Colony (*F*_2,176_ = 32.2, *p* = 0.0001) and species × colony (*F*_2,176_ = 6.00, *p* = 0.0001).

We then performed one-way PERMANOVA as a post hoc test for effects with more than two levels (colony, interaction species × colony).

With regard to the colony effect, all colonies differed significantly among each other (*p* > 0.05), except for the following pairs (colony codes—see Table 1): LIM-RAS, BRW-KAR (one-way PERMANOVA, both *p* = 1.0), OSI-SAM, DZD-KAR (both *p* = 0.99), LIM-SAM (*p* = 0.77), BRW-DZD (*p* = 0.54), BRW-LIM (*p* = 0.22), RAS-SAM (*p* = 0.07) and LIM-OSI (*p* = 0.056).

For the species × colony interaction effect, one-way PERMANOVA indicated that all combinations differed significantly among each other (*p* > 0.05), except for the following combinations:

1. For GCM: SAM-LIM, SAM-OSI (both *p* = 1.0) and OSI-LIM (*p* = 0.08),
2. For GHR: RAS-LIM (*p* = 0.16).

The SIMPER analysis showed that Al, Zn, Mn, Ni, Sr and Fe contributed the most (18%, 11%, 11%, 11%, 11% and 10% respectively) to the pattern of inter-species and inter-colony dissimilarity observed in elemental concentrations (Table S4). Al, Ni and Mn contributed the most (21%, 14% and 11% respectively) to the pattern of inter-species dissimilarity observed in elemental concentrations (Table S4).

### Factors affecting concentrations of particular elements: the effect of species

Univariate PERMANOVA analyses performed separately for particular elements revealed that species significantly affected the levels of all elements (*p* < 0.003) except for Zn (*p* = 0.20). Univariate PERMANOVA analyses performed separately for particular elements showed that colony significantly affected the levels of all elements (all *p* < 0.007), except for As (*p* = 0.14), Pb (*p* = 0.10) and V (*p* = 0.06). The species × colony interaction significantly affected the levels of all the elements (all *p* < 0.044) except for V (*p* = 0.55) (Table S5).

We then performed one-way PERMANOVA as a post hoc test for effects of species, interaction species × colony).

With regard to the species effect, we found significantly higher concentrations of Cd, Mn, Mo, Ni and Sr in eggshells of GCM compared to GHR; the pattern for Al, As, Ca, Cr, Cu, Fe, Hg, Mg, Pb, Se and V was the opposite, with higher concentrations in GHR (Table 3).

**Table 3:** Elemental concentrations (medians and min-max (mg kg^−1^ dw)) in post-hatched eggshells of great cormorant and grey heron

Element	Grey heron (*N* = 98)			Great cormorant (*N* = 94)		
	Median	Min	Max	Median	Min	Max
Al	7.54	0.11	36.31	0.90	0.03	7.11
As	0.42	0.00	1.51	0.11	0.00	0.50
Ca	342,933.5	279,829.9	381,054.2	275,679.2	262,197.5	308,321.9
Cd	0.01	0.00	0.05	0.02	0.00	0.04
Cr	0.14	0.00	0.69	0.13	0.03	0.24
Cu	1.46	0.66	3.70	0.81	0.48	1.70
Fe	6.78	1.73	26.41	3.37	1.07	23.99
Hg	0.13	0.00	0.74	0.09	0.01	1.49
Mg	1114.2	744.9	1554.8	834.4	634.4	1306.0
Mn	1.24	0.30	16.17	3.56	0.70	23.67
Mo	0.02	0.00	0.19	0.08	0.00	0.16
Ni	0.12	0.00	1.33	2.17	0.90	5.04
Pb	0.54	0.10	0.93	0.43	0.00	1.22
Se	0.73	0.00	3.70	0.70	0.16	1.27
Sr	93.35	28.99	265.19	134.83	49.10	295.19
V	0.05	0.00	0.30	0.04	0.00	0.11
Zn	7.85	1.21	35.07	9.12	1.74	39.29

For the species × colony interaction effect in the univariate analyses, we focused on two components:

1. GCM vs GHR differences in particular colonies; the results are presented below,
2. Inter-colony differences separately for GCM and GHR; the results are presented in the Electronic Supplementary Materials.

We found the following patterns of GCM vs GHR differences in particular element concentrations in the colonies:

- Mo: no significant differences (all *p* > 0.21)
- Al: differences among all colonies (all *p* = 0.012–0.024) with higher values in GHR
- As: BRW, DZD, LIM, RAS and SAM (all *p* = 0.012–0.024) with higher values in GHR (Fig. S1)
- Ca: differences among all colonies (all *p* = 0.012) with higher values in GHR (Fig. S1)
- Cd: GAK with higher values in GCM, and SAM with higher values in GHR (both *p* = 0.012) (Fig. S2)
- Cr: BRW, KAR, LIM with higher values in GHR and OSI with higher values in GCM (all *p* = 0.012–0.036) (Fig. S2)
- Cu: BRW, GAK, KAR, LIM with higher values in GHR and SAM with higher values in GCM (all *p* = 0.012–0.024) (Fig. S2)
- Fe: GAK, KAR, RAS with higher values in GHR (all *p* = 0.012–0.024) (Fig. S3)
- Hg: LIM and RAS with higher values in GHR (all *p* = 0.012–0.036) (Fig. S3)
- Mg: BRW, DZD, GAK, KAR, OSI and RAS (all *p* = 0.012) with higher values in GHR (Fig. S3)
- Mn: BRW, DZD, GAK, LIM, OSI and SAM with higher values in GCM (all *p* = 0.012–0.048) (Fig. S4)
- Ni: differences among all colonies with higher values in GCM (all *p* = 0.012) (Fig. S4)
- Pb: GAK with higher values in GHR (*p* = 0.04) (Fig. S5)
- Se: RAS and SAM with higher values in GCM (both *p* = 0.012) (Fig. S5)
- Sr: BRW, RAS and SAM with higher values in GCM (all *p* = 0.012) (Fig. S5)
- Zn: DZD with higher values in GCM and LIM with higher values in GHR (both *p* = 0.012) (Fig. S6).

### Elemental concentrations in industrialised and non-industrialised areas

We found significantly higher concentrations of Fe, Mg, Mn in GCM and GHR eggshells collected in colonies located in industrialised areas compared to non-industrialised areas (Table 4). In GCM, we found significantly higher levels of Ca, Cr, Cu, Ni, Sr and Zn in eggshells collected in colonies situated in industrialised areas (Table 4).

**Table 4:** Elemental concentrations (medians and min-max [mg kg^−1^ dw]) in post-hatched eggshells of great cormorant (GCM) and grey heron (GHR) breeding in colonies in industrialised and non-industrialised areas. Elements with significant differences (*p* < 0.05) are shown in italics. LOD—values below the limit of detection

Species		Industrialised colonies			Non-industrialised colonies			*U* test	
		Median	Min	Max	Median	Min	Max	*Z*	*p*
GCM	Al	0.90	0.07	7.11	0.90	0.03	4.74	0.05	0.96
	As	0.10	LOD	0.30	0.12	LOD	0.50	− 0.41	0.68
	*Ca*	287,704	263,665	308,322	274,659	LOD	287,030	3.61	*< 0.001*
	Cd	0.02	LOD	0.03	0.02	LOD	0.04	− 1.05	0.29
	*Cr*	0.15	0.07	0.24	0.12	0.03	0.24	1.96	*0.0496*
	*Cu*	1.04	0.62	1.70	0.74	0.48	1.09	6.18	*< 0.001*
	*Fe*	6.05	2.08	15.82	2.63	1.07	23.99	5.97	*< 0.001*
	Hg	0.09	0.03	0.76	0.08	0.01	1.49	0.44	0.66
	*Mg*	857	682	985	809	634	1306	2.40	*0.02*
	*Mn*	6.42	1.75	23.67	2.82	0.70	13.44	4.17	**<** *0.001*
	Mo	0.08	LOD	0.15	0.08	LOD	0.16	0.17	0.87
	*Ni*	2.59	1.31	5.04	1.97	0.90	3.92	2.30	*0.02*
	Pb	0.47	0.00	1.22	0.41	LOD	1.06	0.78	0.43
	Se	0.72	0.44	1.03	0.65	0.16	1.27	1.69	0.09
	*Sr*	152	68	295	107.8	49.1	232.8	2.18	*0.029*
	V	0.04	LOD	0.11	0.04	LOD	0.10	− 0.24	0.81
	*Zn*	11.82	6.23	39.29	7.49	1.74	13.86	5.97	*< 0.001*
GHR	Al	7.29	3.18	17.91	7.68	0.11	36.31	0.44	0.66
	As	0.42	0.00	0.94	0.42	LOD	1.51	− 0.08	0.93
	Ca	344,333	334,776	381,054	342,582	279,830	377,697	0.84	0.40
	Cd	0.01	LOD	0.04	0.01	LOD	0.05	− 1.47	0.14
	Cr	0.11	LOD	0.69	0.14	LOD	0.65	− 0.18	0.85
	Cu	1.36	0.74	3.69	1.50	0.66	3.49	− 1.21	0.23
	*Fe*	7.76	4.07	19.27	4.76	1.73	26.41	3.85	*< 0.001*
	Hg	0.12	0.05	0.34	0.13	LOD	0.74	− 0.53	0.59
	*Mg*	1170	890	1555	1081	745	1441	2.78	*0.005*
	*Mn*	1.49	0.61	16.17	1.14	0.30	2.68	2.41	*0.02*
	Mo	0.01	LOD	0.12	0.03	LOD	0.19	− 1.00	0.32
	Ni	0.12	LOD	0.28	0.13	0.03	1.33	− 1.46	0.14
	Pb	0.53	0.23	0.88	0.54	0.10	0.93	0.07	0.94
	Se	0.79	0.16	2.72	0.70	LOD	3.70	0.18	0.85
	Sr	88.49	36.46	223.18	97.25	28.99	265.19	0.20	0.84
	V	0.04	LOD	0.25	0.07	LOD	0.30	− 1.44	0.15
	Zn	8.89	2.93	31.81	7.25	1.21	35.07	1.01	0.31

## Discussion

To the best of our knowledge, this is the first study investigating the concentrations of multiple elements in the eggshells of sympatrically nesting great cormorants (GCM) and grey herons (GHR) in Europe. Investigation of contamination levels in tissues and eggs of these top predators in freshwater ecosystems is an important aspect of monitoring the health of aquatic habitats.

### Possible common sources of elements

As an organism can absorb elements in many different ways (food, water, atmosphere), it is practically impossible to identify the sources of particular elements in it. However, a high degree of clustering or the correlation of particular elements in multivariate analyses like HACA or PCA may help to identify possible common sources of elements (Hashmi et al. 2013; Kitowski et al. 2018).

The common clustering of Ca–Mg–Sr in HACA may reflect the frequent substitution of these limestone elements in geochemical and physiological processes in an organism (MacMillan et al. 2002). They may be absorbed by a female from bones of fish which contain high levels of limestone elements (Radwan et al. 1990; Sharif et al. 1993; Torz and Nedzarek 2013). The eggshell serves as the major source of both Ca and Mg for the developing embryo (Richards and Packard 1996; Orlowski et al. 2014, 2016). Biochemically, Sr is very similar to Ca, which may result in enzymatic or structural substitutions during nutrient uptake (Mora et al. 2003; Matz and Rocque 2007). As a limestone element, Sr is very similar to Ca and may be mobilised from maternal rocks (Bielanski 1996; Kabata-Pendias and Mukherjee 2007; Mora et al. 2011). Post-hatching eggshells are depleted in these elements as they are transferred to the developing chick. Thus, their common clustering in HACA may indicate a common outflow rather than a common source.

The clusters Cd–V–Cr–Hg–Mo–As in GCM and Hg–Ni in GHR (Fig. 3) are probably due to agrochemical runoff from arable lands to lakes and rivers; Cd and As often originate from agrochemicals (Nicholson et al. 2003; Nziguheba and Smolders 2008). Fertilisers have been identified as a source of soil Hg contamination (Mortvedt 1995; Otero et al. 2005). Sewage sludge and some P fertilisers have been recognised as important sources of Ni in agricultural soils (Kabata-Pendias and Szteke 2015). The application of chemical fertilisers may also change the speciation and mobility of heavy metals (Cu, Cr and Ni) in the soil (Liu et al. 2007), increasing their availability to soil invertebrates that are components of the GHR’s diet. Moreover, water bodies in industrialised areas are rich in Cd, Cr, Hg and Ni (Kabata-Pendias and Mukherjee 2007).

We also found distinctive clusters characteristic only of GCM (Fe–Mn–Zn–Ni) or GHR (Fe–Al–Zn). The former may have originated from bottom sediments, as all these elements have a strong tendency to accumulate in sediments (Barbusinski and Nocon 2011; Rzetala et al. 2013). The Fe–Al–Zn cluster, found exclusively in GHR, may have originated from parent rocks or pollutants dissolved in water. Zn can enter river systems from numerous sources, such as mine drainage, industrial and municipal wastes, urban runoff and soil erosion waters (Kabata-Pendias and Pendias 2010). Natural and mineral fertilisers are an important source of the total annual inputs of Zn into agricultural soils (Nicholson et al. 2003; Nziguheba and Smolders 2008). Alternatively, these clusters may represent the input of these elements from fish containing high levels of Fe, Ca, Zn and Mg (Radwan et al. 1990; Luczynska et al. 2009).

The clusters Cu-Se-Al-Pb in GCM and Cu-Mn-Se-As-Pb in GHR may represent input from aquatic organisms rich in Cu, Se and Mn (Radwan et al. 1990; Elder and Collins 1991; Luczynska et al. 2009; Burghelea et al. 2011) and/or pollutants from herbicides and insecticides and fertilisers rich in Pb and As (Mandal and Suzuki 2002; Nziguheba and Smolders 2008; Jiao et al. 2012) and/or hard coal excavation, processing and combustion emitting pollution rich in Mn, As and Pb (Pasieczna et al. 2010; Nocon 2006; Barbusinski and Nocon 2011; Juda-Rezler and Kowalczyk 2013; Smolka-Danielowska 2015). The higher concentrations of Cr, Fe, Mn, Ni and Zn in the eggshells of GCM and Fe and Mn in those of GHR from colonies situated in industrialised areas suggest that these elements may be anthropogenic.

### Inter-group differences

We found that Al, Ni and Mn contributed the most (21%, 14% and 11%, respectively) to the pattern of inter-species dissimilarity observed in elemental concentrations.

The eggshells of GHR had significantly higher concentrations of Al compared to GCM, which can be explained in the context of differences in stomach pH. Herons have an extremely efficient digestive system (Vinokurov 1960) with a low pH (2.5–4.9) (Mennega 1938). In consequence, GHR pellets, in contrast to those of GCM, rarely contain any remains of fish, despite the fact that this type of prey is an important part of the diet (Jakubas and Mioduszewska 2005). The low pH in the GHR stomach favours the release of Al compounds. As high levels of some Al compounds may cause DNA damage (Kabata-Pendias and Pendias 2010), females may sequester this element to eggshells. Stomach pH values (3.9–6.3) reported for GCM (Gremillet et al. 2000) probably do not favour ingestion of Al compounds as their solubility is the lowest in the pH range 4.5–9.5 (Rosseland et al. 1990; Barabasz et al. 2002; Goworek 2006).

We found that Ni was the second element contributing the most to inter-species dissimilarity. The eggshells of GCM had significantly higher concentrations of this element compared to GHR. Results of HACA suggest different sources of Ni in both species. Clustering with Hg in GHR may indicate agrochemical runoff from arable lands to lakes and rivers as fertilisers have been identified as a source of soil Hg and Ni contamination (Mortvedt 1995; Otero et al. 2005; Kabata-Pendias and Szteke 2015). Clustering with Fe, Mn and Zn in GCM suggests transfer from bottom sediments. Ni does not remain long in aquatic environments as soluble species, because it is easily adsorbed by the suspended matter and Fe–Mn hydroxides, and is deposited in bottom sediments (Muyssen et al. 2004; Szarek-Gwiazda et al. 2011; Barbusinski and Nocon 2011; Rzetala et al. 2013; Kabata-Pendias and Szteke 2015). Thus, GCM foraging on demersal fish may be prone to Ni accumulation. The lower concentrations of this element in the eggshells of GHR from the same colonies can be explained by the foraging of this species in a wider spectrum of habitats and microhabitats (only the littoral zones of water bodies) compared to GCM. Accordingly, we found higher levels of Ni in the eggshells of GCM breeding in industrialised areas, recording the highest concentrations of this element at DZD, in the highly polluted region of upper Silesia. Ni is broadly used in several industries and is considered as a serious pollutant, that is, released from metal-processing plant and from the combustion of coal and oil (Kabata-Pendias and Szteke 2015). Bottom sediments may be contaminated by Ni from mining and smelting wastewaters. The polluted sediments of the Dzierżno Duże reservoir, an important foraging area of GCM breeding at DZD, contain 51.8 mg kg^−1^ of Ni (Rzetala 2016).

As we had expected, Mn concentrations in GCM eggshells were significantly higher compared to GHR. This may be explained by the obligate piscivorous diet of GCM vs facultative piscivorous diet in GHR. This element can be significantly bioconcentrated by aquatic biota, in that fish (estimated bioconcentration factor = 35–930) (Howe et al. 2004), with tissues rich in this element (liver 1.1–19.0 mg kg^−1^ dw, bones 30.0–88.5 mg kg^−1^ dw) (Radwan et al. 1990). Inter-species difference in Mn concentration can also be interpreted in terms of different foraging tactics. GCMs preying on fish foraging on demersal organisms are exposed to Mn accumulated in contaminated sediments (Howe et al. 2004; Czaplicka et al. 2016). In water, Mn compounds and species are easily transferred into colloidal forms and precipitated in bottom sediments (Kabata-Pendias and Szteke 2015). The significant clustering of Mn and Fe in HACA found only for GCM (Fig. 3a) suggests the transfer of these elements from sediments to demersal fish. The highest concentrations of Mn (and also Fe) recorded in the eggshells of GCM breeding at DZD are attributable mainly to the inflow of mine waters from hard coal mines containing elevated concentrations of Fe and Mn (Choinski 2006; Barbusinski and Nocon 2011) to the Dzierżno Duże reservoir, an important foraging area for birds from that colony. In both studied species, we found higher levels of Mn in the eggshells of individuals breeding in industrialised areas. This element is also widely used in industry, especially in metallurgy; municipal wastewater, sewage sludge, and metal smelting processes are considered as major anthropogenic sources of Mn (Kabata-Pendias and Szteke 2015). Also significantly higher levels of Fe in eggshells of both species breeding in industrialised areas may be explained by common use of this element in industry. Dissolved Fe compounds readily precipitate in aquatic environments forming various multimetallic concretions in bottom sediments (Kabata-Pendias and Szteke 2015).

Contrary to our expectations, Cu, Se and Hg levels were significantly lower in eggshells of piscivorous GCM compared to the more opportunistic forager, GHR. This can be explained by the latter’s wider dietary spectrum: this includes aquatic organisms like water beetles, snails and frogs, which accumulate relatively high levels of all three elements (Eisler 1997; Loumbourdis and Wray 1998; Bergeron et al. 2010). However, the median values of Hg (mg kg^−1^ dw) recorded in GHR (0.13) were lower than those reported for GHR eggshells from colonies along the Rivers Odra and Vistula (0.31–0.34) (Dmowski 1999). This may indicate a decrease in Hg contamination of aquatic ecosystems compared to the 1990s: this has been suggested by other studies on waterbirds from Poland (Kalisinska et al. 2014; Kitowski et al. 2015). We found significant clustering of Cu and Se only for GCM (Fig. 3a); this suggests a common source, i.e. fish, of these elements for this species, and but various sources of those elements for GHR (Fig. 3b), including fish but also other aquatic organisms or even rodents (Giles 1981; Jakubas and Mioduszewska 2005; Jakubas and Manikowska 2011).

Contrary to our expectations, regarding the Pb and Cd concentration patterns (i.e. higher concentrations in industrialised areas), we found no inter-colony differences for GCM and just a few differences for GHR. Pb levels in GHR eggshells collected at KAR were higher than at SAM. The spatial variation of Pb and Cd concentrations in GHR eggshells and its absence with regard to GCM suggest that non-aquatic sources of this element are important. GHR from the KAR colony also forage on farmland (Zulawy Wislane—the large alluvial plain in the Vistula delta with extensive agricultural land) in the vicinity of the colony (Jakubas D.—unpublished data), where they are exposed to Pb and Cd contamination from soil invertebrates accumulating large amounts of these elements from mineral fertilisers (Stone et al. 2002; Carpene et al. 2006; Purchart and Kula 2007; Nziguheba and Smolders 2008), directly by hunting for them or indirectly from ingested predators of organisms bioaccumulating this element. In this context, the highest Cd concentration in GHR eggshells, recorded at LIM and SAM in the Masurian Lake District, can be attributed to runoff of Cd from fertilised soils to water bodies.

We recorded significantly higher concentrations of Zn in GCM eggshells from industrialised areas. Anthropogenic Zn sources are related to several industrial processes and agricultural practices. The largest discharge of this element to aquatic environments in the European Union countries is from the manufacturing of basic industrial chemicals (Kabata-Pendias and Szteke 2015). In GCM eggshells, the highest levels were recorded at RAS, BRW and DZD. The first two of these colonies are influenced by rivers with high Zn levels accumulated in the sediments (up to 2000 mg kg^−1^ in the Vistula and 14,000 mg kg^−1^ in the Odra; Kabata-Pendias and Szteke 2015); the Vistula carries an annual amount of 30.77 t of Zn to the Baltic Sea (Central Statistical Office 2015). In addition, the sediments of the Dzierżno Duże reservoir are reported to contain considerable Zn levels (895.6 mg kg^−1^) (Rzetala 2016). This therefore suggests that riverine and reservoir sediments are the main source of Zn in GCM eggshells from the RAS, BRW and DZD colonies.

Cr concentrations were significantly higher in GCM eggshells from industrialised areas. Various industry sectors commonly use Cr compounds in dyes, paints and superalloys and its compounds are often found in soil and groundwater (Kimbrough et al. 1999; Faisal and Hasnain 2006). In GHR, the highest Cr levels were recorded in eggshells from the colonies at KAR and BRW, where the birds foraged in areas influenced by the Vistula River (Vistula Lagoon, Wloclawek Reservoir). The Vistula carries 10.4–11.3 t of Cr to the Baltic Sea annually (Central Statistical Office 2013, 2015). Cr compounds are very persistent in sediments, and aquatic plants, fish and invertebrates are capable of accumulating large amounts of this element (plants up to 1000 mg kg^−1^) (Kabata-Pendias and Szteke 2015).

### Limitations of our study

We are aware that our study has limitations. Firstly, our interpretations of the observed differences in elemental concentrations focus mainly on dietary differences in local soil and water pollution sources. However, many other factors, such as metabolic state and health can also affect the sequestration of particular elements into eggs. Secondly, we have no data on elemental concentrations in potential food or in the environment from the season and areas studied; our information is based on literature data. Thirdly, as this study is correlational, it is not possible to indicate the actual sources of elements in the eggshells. Nevertheless, this study provides recent data on elemental concentrations in the eggshells of two top predators in aquatic ecosystems, regarded as important ‘indicator species’ in environmental monitoring. As non-essential metals are sequestered into the eggshell for excretion, the post-hatched eggshells, easy to collect in breeding colonies of waterbirds may provide a convenient, non-invasive tool for monitoring heavy metals contaminations in this group of birds (Lam et al. 2005; Ayas 2007; Kitowski et al. 2018). In order to gain a broader picture of contamination levels in these two species, future studies should also examine egg contents, and the tissues of chick and adult birds.

## Conclusions

We found some variation in elemental concentrations in eggshells of two waterbirds breeding sympatrically in mixed colonies. The observed inter-species variations were due to differences in digestion (Al) and the proportion of fish and other organisms in the diet (Cu, Mn). Inter-colony differences were attributed to local sources of pollution. The higher levels of some elements in the eggshells of great cormorants (Fe, Mn, Cr, Ni, Zn) and grey herons (Fe, Mn) breeding in industrialised areas may be a sign of the negative environmental impact of large industrial plants.

## Electronic supplementary material

ESM 1 (DOC 330 kb)
