# Supplementary material for: Inter-species and inter-colony differences in elemental concentrations in eggshells of sympatrically nesting great cormorants Phalacrocorax carbo and grey herons Ardea cinerea
Source: Environ Sci Pollut Res Int. 2018 Nov 27;26(3):2747–60. doi: 10.1007/s11356-018-3765-5 (PMC6338717; doi:10.1007/s11356-018-3765-5)
Supplement: Supplementary file 1 — (DOC 330 kb) [file 11356_2018_3765_MOESM1_ESM.doc]

Electronical supplementary information

Inter-species and inter-colony differences in elemental concentrations in eggshells of sympatrically nesting Great Cormorants Phalacrocorax *carbo* and Grey Herons *Ardea cinerea*

Dariusz Jakubas, Ignacy Kitowski, Dariusz Wiącek, Szymon Bzoma

Univariate PERMANOVA analyses performed separately for particular elements revealed that Species x Colony interaction affected significantly elemental concentrations in eggshells. We present here results of inter-colony (colony codes – see Table 1) variation for particular species, i.e. Great Cormorants and Grey Herons. We found the following patterns:

1) in Great Cormorants:

**As, Cd, Cr,** **Mo** – no significant differences (all *p* = 1.0),

**Mg** – no significant differences (all *p* = 0.18-1.0),

**Pb** – no significant differences (all *p* = 0.46-1.0),

**Se** – no significant differences (all *p* = 0.13-1.0).

**Al** RAS-OSI (*p* = 0.048) with higher value in OSI (Table ES2, Fig. ES1).

**Ca** – diffrences among all colonies (*p* = 0.012-0.036) except for GAK-DZD, LIM-KAR, SAM-KAR, SAM-LIM (all *p* = 1.0) with highest values at BRW and lowest at OSI (Table ES2, Fig. ES1).

**Cu** - GAK-BRW, KAR-BRW, KAR-LIM, LIM-BRW, OSI-BRW, OSI-GAK, RAS-GAK, RAS-KAR, RAS-LIM, SAM-BRW, SAM-RAS (*p* = 0.012-0.036) with the highest values at BRW and RAS (Table ES2, Fig. ES2).

**Fe** - GK-BRW, GAK-DZD, KAR-DZD, OSI-BRW, OSI-DZD, RAS-DZD, SAM-BRW, SAM-DZD (*p* = 0.012-0.024) with the highest values at DZD (Table ES2, Fig. ES3).

**Hg** – SAM-GAK, SAM-KAR,SAM-OSI, SAM-RAS (*p* = 0.012-0.48) with the highest values at SAM (Table ES2, Fig. ES3).

**Mn** – GAK-BRW, GAK-DZD, KAR-BRW, KAR-DZD (*p* = 0.012-0.024) with the highest values at DZD (Table ES2, Fig. ES4).

**Ni** – DZD-BRW, LIM-BRW, LIM-DZD, LIM-GAK, LIM-KAR, OSI-DZD, OSI-GAK, RAS-BRW, RAS-DZD, RAS-GAK, SAM-BRW, SAM-DZD, SAM-GAK, SAM-KAR (*p* = 0.012-0,048) with the highest values at DZD (Table ES2, Fig. ES4).

**Sr** – LIM-BRW, LIM-DZD, LIM-GAK, OSI-DZD, OSI-GAK, OSI-KAR, RAS-BRW, RAS-DZD, RAS-GAK, RAS-KAR, SAM-BRW, SAM-DZD, SAM-GAK, SAM-KAR (*p* = 0.012- 0.36) with the highest values at DZD and GAK (Table ES2, Fig. ES5).

**Zn** - GAK-BRW, GAK-DZD, KAR-GAK, LIM-GAK, OSI-DZD, OSI-GAK, OSI-LIM, RAS-GAK, RAS-KAR, RAS-LIM, RAS-OSI, SAM-GAK, SAM-RAS (all *p* = 0.012) with the highest values at RAS and smallest at GAK (Table ES2, Fig. ES6).

2) in Grey Herons we found the following significant differences (*p* < 0.05):

**Al** – GAK-BRW, GAK-DZD, GAK-KAR, KAR-LIM, OSI-GAK, RAS-BRW, RAS-GAK, RAS-KAR, RAS-OSI, SAM-KAR, SAM-OSI (*p* = 0.012-0.036) with highest values at KAR and lowest at GAK (Table ES3, Fig. ES1).

**As** - LIM-DZD, LIM-GAK, RAS-DZD, SAM-BRW, SAM_DZD, SAM_GAK, SAM-KAR, SAM-OSI (*p* = 0.012-048) with the highest values at SAM (Table ES3, Fig. ES1).

**Ca** - BRW-GAK (*p* = 0.024) with the highest value at BRW (Table ES3, Fig. ES1).

**Cd** - DZD-BRW, GAK-BRW, KAR-DZD, KAR-GAK, LIM-BRW, LIM-DZD, LIM-GAK, LIM-KAR, OSI-KAR, OSI-LIM, RAS-DZD, RAS-GAK, SAM-BRW, SAM-DZD, SAM-GAK,SAM-KAR, SAM-OSI (*p* = 0.012-0.24) with the highest vales at SAM and LIM (Table ES3, Fig. ES2).

**Cr** - DZD-BRW, GAK-BRW, KAR-DZD, KAR-GAK, LIM-BRW, LIM-DZD, LIM-KAR, OSI-BRW, OSI–KAR, RAS-BRW, RAS-KAR SAM-BRW, SAM-KAR (*p* = 0.012-0.024) with the highest values in BRW and KAR (Table ES3, Fig. ES2).

**Cu** - DZD-BRW, GAK-BRW, KAR-BRW,KAR-DZD, KAR-GAK, LIM-BRW, LIM-KAR, OSI-BRW, OSI-KAR, RAS-BRW, RAS-KAR, SAM-BRW, SAM-KAR (all *p* = 0.012) with the highest values at BRW and KAR (Table ES3, Fig. ES2).

**Fe** - GAK-BRW, KAR-GAK, OSI-BRW, OSI-DZD, OSI-KAR, RAS-GAK, RAS-OSI, SAM-BRW, SAM-DZD, SAM-KAR, SAM-LIM, SAM-RAS (*p* = 0.012-0.048) with the lowest values at OSI and SAM (Table ES3, Fig. ES3).

**Hg** – LIM-DZD, LIM-GAK, RS-DZD, LIM-GK, SAM-DZD, SAM-GAK (*p* = 0.012-0.036) with the highest values at LIM and SAM (Fig. ES3).

**Mg** – LIM-BRW, LIM-DZD, LIM-GAK, LIM-KAR, RAS-BRW, SAM-BRW, SAM-DZD, SAM-GAK, SAM-KAR, SAM-OSI, SAM-RAS (*p* = 0.012-0.036) with the lowest values at LIM and SAM (Table ES3, Fig. ES3).

**Mn** – OSI-BRW, OSI-LIM, RAS-OSI (*p* = 0.012-0.036) with the lowest values at OSI (Table ES3, Fig. ES4).

**Mo** - DZD-BRW, KAR-DZD, KAR-GAK, LIM-BRW, LIM-KAR, OSI-BRW, OI-KAR, RAS-BRW, RAS-KAR, SAM-KAR (*p* = 0.012-0.024) with the highest values at KAR (Table ES3, Fig ES4).

**Ni** – DZD-BRW, GAK-DZD, KAR-DZD, LIM-DZD, OSI-DZD, RS-DZD, SAM-BRW, SAM-DZD, SAM-LIM, SAM-RAS (*p* = 0.012-0.048) with the lowest values at DZD (Table ES3, Fig. ES4).

**Pb** - SAM-KAR (*p* = 0.012) with the highest value at KAR (Table ES3, Fig. ES5).

**Se** - RAS-BRW, RS-DZD, RAS-GAK, RAS-KAR, RAS-OSI, SAM-BRW, SAM-DZD, SAM-GAK, SAM-KARSAM-OSI (*p* = 0.012-0.024) with the highest values at RAS and SAM (Table ES3, Fig. ES5).

**Sr** – DZD-BRW, GAK-BRW, GAK-DZD, KAR-BRW, LIM-BRW, LIM-DZDZ, LIM-GAK, LIM-KAR, OSI-DZD, OSI-GAK, OSI-KAR, RAS-BRW, RAS-DZD, RAS-GAK, RAS-KAR, SAM-BRW, SAM-DZD, SAM-GAK, SAM-KAR (*p* = 0.012-0.048) with the highest values at DZD, GAK and KAR (Table ES3, Fig. ES5).

**Zn** - DZD-BRW, GAK-BRW, KAR-DZD, KAR-GAK, LIM-BRW, LIM-DZD, LIM-GAK, LIM-KAR, OSI-LIM, RAS-BRW, RAS-DZD, RAS-GAK, RAS-KAR, RAS-OSI (*p* = 0.012-0.048) with the highest values at LIM and RAS (Table ES3, Fig. ES6).

Table ES1. Validation of the analytical method: linearity (the ability of the method to obtain test results proportional to the concentration of the analyte; *r* - Pearson correlation coefficient, detection limit and recoveries for the studied elements.

| Element | Linearity *r* | Limit of detection  LOD [µg/L] | Recovery  [%] |
| --- | --- | --- | --- |
| As | 0.9995 | 0.011 | 99 |
| Ca | 0.9985 | 0.002 | 105 |
| Cd | 0.9999 | 0.001 | 97 |
| Cr | 0.9997 | 0.003 | 97 |
| Cu | 0.9999 | 0.002 | 103 |
| Fe | 0.9998 | 0.021 | 96 |
| Hg | 0.9996 | 0.058 | 97 |
| Mg | 0.9953 | 0.005 | 104 |
| Mn | 0.9998 | 0.002 | 96 |
| Mo | 0.9996 | 0.022 | 98 |
| Ni | 0.9999 | 0.001 | 97 |
| Pb | 0.9999 | 0.010 | 98 |
| Sc | 0.9997 | 0.002 | 99 |
| Se | 0.9995 | 0.012 | 97 |
| Sr | 0.9998 | 0.003 | 98 |
| V | 0.9999 | 0.003 | 97 |
| Zn | 0.9998 | 0.010 | 102 |

Table ES2. Concentrations of all elements in eggshells of Great Cormorants in all studied colonies. Med – median, Species codes – see Table 1.

|  | OSI |  |  | KAR |  |  | DZD |  |  | GAK |  |  | BRW |  |  | LIM |  |  | RAS |  |  | SAM |  |  |
| --- | --- | --- | --- | --- | --- | --- | --- | --- | --- | --- | --- | --- | --- | --- | --- | --- | --- | --- | --- | --- | --- | --- | --- | --- |
|  | Med | Min | Max | Med | Min | Max | Med | Min | Max | Med | Min | Max | Med | Min | Max | Med | Min | Max | Med | Min | Max | Med | Min | Max |
| Al | **1.34** | 0.43 | 1.57 | **0.56** | 0.31 | 1.45 | **1.58** | 0.11 | 7.11 | **0.89** | 0.03 | 1.37 | **1.02** | 0.07 | 3.61 | **0.60** | 0.08 | 4.74 | **0.29** | 0.09 | 0.95 | **1.29** | 0.07 | 1.93 |
| As | **0.12** | 0.00 | 0.38 | **0.07** | 0.00 | 0.50 | **0.08** | 0.00 | 0.28 | **0.02** | 0.00 | 0.44 | **0.10** | 0.00 | 0.30 | **0.14** | 0.00 | 0.30 | **0.11** | 0.00 | 0.30 | **0.14** | 0.00 | 0.29 |
| Ca* | **267** | 262 | 268 | **276** | 271 | 282 | **269** | 264 | 274 | **272** | 264 | 275 | **301** | 286 | 308 | **280** | 270 | 287 | **288** | 284 | 293 | **278** | 272 | 283 |
| Cd | **0.03** | 0.00 | 0.03 | **0.02** | 0.00 | 0.04 | **0.01** | 0.00 | 0.02 | **0.02** | 0.00 | 0.04 | **0.02** | 0.00 | 0.03 | **0.02** | 0.01 | 0.03 | **0.02** | 0.00 | 0.03 | **0.02** | 0.00 | 0.03 |
| Cr | **0.11** | 0.05 | 0.19 | **0.15** | 0.06 | 0.19 | **0.15** | 0.08 | 0.24 | **0.13** | 0.07 | 0.19 | **0.15** | 0.07 | 0.20 | **0.12** | 0.07 | 0.22 | **0.15** | 0.08 | 0.23 | **0.13** | 0.03 | 0.24 |
| Cu | **0.85** | 0.64 | 1.09 | **0.72** | 0.52 | 0.81 | **0.86** | 0.62 | 1.50 | **0.64** | 0.48 | 0.81 | **1.12** | 0.88 | 1.46 | **0.72** | 0.56 | 0.86 | **1.13** | 0.85 | 1.70 | **0.81** | 0.63 | 1.03 |
| Fe | **1.84** | 1.33 | 3.36 | **3.29** | 2.03 | 7.49 | **7.81** | 3.73 | 15.82 | **1.93** | 1.07 | 5.75 | **6.03** | 3.38 | 8.13 | **3.41** | 2.41 | 23.99 | **3.93** | 2.08 | 6.72 | **2.26** | 1.33 | 5.80 |
| Hg | **0.08** | 0.01 | 0.17 | **0.07** | 0.02 | 0.15 | **0.08** | 0.03 | 0.21 | **0.07** | 0.02 | 0.13 | **0.19** | 0.06 | 0.76 | **0.09** | 0.02 | 0.17 | **0.05** | 0.03 | 0.18 | **0.17** | 0.08 | 1.49 |
| Mg | **809** | 671 | 1081 | **800** | 708 | 918 | **826** | 682 | 923 | **796** | 634 | 934 | **896** | 800 | 963 | **827** | 702 | 1306 | **860** | 732 | 985 | **786** | 699 | 990 |
| Mn | **3.17** | 0.70 | 7.98 | **1.61** | 1.00 | 4.82 | **9.30** | 2.67 | 23.67 | **2.28** | 1.20 | 5.49 | **6.99** | 3.17 | 11.86 | **3.85** | 1.16 | 6.76 | **2.78** | 1.75 | 6.49 | **4.52** | 1.21 | 13.44 |
| Mo | **0.10** | 0.00 | 0.14 | **0.07** | 0.00 | 0.12 | **0.06** | 0.00 | 0.14 | **0.06** | 0.00 | 0.13 | **0.05** | 0.00 | 0.14 | **0.05** | 0.00 | 0.16 | **0.11** | 0.00 | 0.15 | **0.09** | 0.01 | 0.15 |
| Ni | **1.83** | 1.19 | 2.52 | **3.03** | 1.13 | 3.64 | **3.84** | 2.53 | 5.04 | **2.94** | 2.30 | 3.92 | **2.59** | 1.69 | 3.02 | **1.39** | 0.90 | 2.32 | **1.53** | 1.31 | 2.17 | **1.43** | 1.30 | 2.22 |
| Pb | **0.47** | 0.19 | 0.90 | **0.48** | 0.00 | 0.83 | **0.62** | 0.00 | 1.06 | **0.31** | 0.12 | 0.69 | **0.51** | 0.00 | 1.22 | **0.38** | 0.07 | 0.91 | **0.35** | 0.00 | 1.09 | **0.38** | 0.10 | 1.06 |
| Se | **0.56** | 0.25 | 0.86 | **0.78** | 0.38 | 1.27 | **0.69** | 0.50 | 1.03 | **0.64** | 0.16 | 1.09 | **0.72** | 0.44 | 0.94 | **0.80** | 0.34 | 1.16 | **0.74** | 0.55 | 0.94 | **0.76** | 0.46 | 0.93 |
| Sr | **87.6** | 65.5 | 112.2 | **175.7** | 79.6 | 232.8 | **222.5** | 140.9 | 295.2 | **162.4** | 122.7 | 226.0 | **157.5** | 116.7 | 182.9 | **75.6** | 49.1 | 149.3 | **76.6** | 67.6 | 85.1 | **89.9** | 61.4 | 145.0 |
| V | **0.05** | 0.01 | 0.06 | **0.03** | 0.00 | 0.10 | **0.05** | 0.00 | 0.11 | **0.05** | 0.00 | 0.09 | **0.02** | 0.00 | 0.11 | **0.03** | 0.00 | 0.07 | **0.04** | 0.00 | 0.08 | **0.03** | 0.00 | 0.10 |
| Zn | **7.32** | 6.29 | 8.16 | **8.71** | 4.68 | 11.03 | **10.96** | 8.36 | 18.30 | **2.54** | 1.74 | 3.32 | **11.04** | 6.23 | 16.65 | **10.12** | 6.98 | 13.86 | **23.45** | 9.40 | 39.29 | **8.75** | 5.39 | 12.39 |

* - x 10^3^

Table ES3. Concentrations of all elements in eggshells of Grey Herons in all studied colonies. Med – median, Species codes – see Table 1.

|  | OSI |  |  | KAR |  |  | DZD |  |  | GAK |  |  | BRW |  |  | LIM |  |  | RAS |  |  | SAM |  |  |
| --- | --- | --- | --- | --- | --- | --- | --- | --- | --- | --- | --- | --- | --- | --- | --- | --- | --- | --- | --- | --- | --- | --- | --- | --- |
|  | Med | Min | Max | Med | Min | Max | Med | Min | Max | Med | Min | Max | Med | Min | Max | Med | Min | Max | Med | Min | Max | Med | Min | Max |
| Al | **12.10** | 9.74 | 15.50 | **16.03** | 4.41 | 36.31 | **6.43** | 3.18 | 17.91 | **3.43** | 3.06 | 5.85 | **12.29** | 5.06 | 16.92 | **7.39** | 0.11 | 17.51 | **6.00** | 5.19 | 11.08 | **4.86** | 3.89 | 15.38 |
| As | **0.31** | 0.00 | 0.78 | **0.35** | 0.00 | 0.60 | **0.41** | 0.17 | 0.48 | **0.31** | 0.00 | 0.52 | **0.38** | 0.18 | 0.73 | **0.82** | 0.20 | 1.08 | **0.77** | 0.00 | 0.94 | **0.82** | 0.35 | 1.51 |
| Ca* | **343** | 334 | 353 | **353** | 332 | 378 | **342** | 335 | 381 | **341** | 322 | 347 | **348** | 343 | 352 | **341** | 280 | 351 | **342** | 335 | 358 | **342** | 332 | 352 |
| Cd | **0.01** | 0.00 | 0.01 | **0.01** | 0.01 | 0.02 | **0.01** | 0.00 | 0.01 | **0.01** | 0.00 | 0.02 | **0.01** | 0.01 | 0.02 | **0.03** | 0.01 | 0.05 | **0.02** | 0.01 | 0.04 | **0.04** | 0.03 | 0.04 |
| Cr | **0.01** | 0.00 | 0.12 | **0.54** | 0.41 | 0.65 | **0.05** | 0.00 | 0.43 | **0.12** | 0.02 | 0.23 | **0.44** | 0.35 | 0.69 | **0.23** | 0.00 | 0.40 | **0.10** | 0.00 | 0.23 | **0.04** | 0.00 | 0.22 |
| Cu | **1.54** | 0.86 | 2.00 | **2.84** | 2.32 | 3.49 | **1.30** | 0.79 | 1.53 | **1.60** | 0.80 | 1.91 | **2.72** | 2.34 | 3.69 | **1.28** | 0.66 | 1.41 | **1.04** | 0.74 | 1.58 | **1.39** | 1.15 | 1.63 |
| Fe | **3.39** | 2.44 | 6.71 | **7.64** | 4.75 | 26.41 | **7.07** | 4.07 | 19.27 | **4.54** | 2.76 | 10.64 | **8.56** | 6.05 | 10.08 | **6.96** | 3.34 | 10.91 | **7.98** | 6.11 | 13.66 | **2.26** | 1.73 | 12.20 |
| Hg | **0.10** | 0.00 | 0.17 | **0.12** | 0.00 | 0.71 | **0.10** | 0.06 | 0.14 | **0.09** | 0.02 | 0.15 | **0.12** | 0.05 | 0.19 | **0.28** | 0.09 | 0.74 | **0.20** | 0.09 | 0.34 | **0.23** | 0.05 | 0.34 |
| Mg | **1124** | 1048 | 1289 | **1180** | 927 | 1441 | **1151** | 1000 | 1319 | **1152** | 1060 | 1349 | **1281** | 1064 | 1555 | **906** | 754 | 1271 | **1034** | 890 | 1226 | **922** | 745 | 976 |
| Mn | **0.57** | 0.34 | 1.28 | **1.64** | 0.75 | 2.68 | **1.03** | 0.61 | 3.61 | **1.11** | 0.46 | 1.94 | **1.59** | 0.92 | 2.76 | **1.59** | 0.78 | 2.59 | **1.99** | 0.96 | 16.17 | **1.01** | 0.30 | 2.41 |
| Mo | **0.00** | 0.00 | 0.03 | **0.09** | 0.04 | 0.19 | **0.01** | 0.00 | 0.05 | **0.02** | 0.00 | 0.10 | **0.07** | 0.02 | 0.12 | **0.00** | 0.00 | 0.19 | **0.00** | 0.00 | 0.10 | **0.02** | 0.00 | 0.10 |
| Ni | **0.13** | 0.09 | 0.16 | **0.20** | 0.03 | 0.27 | **0.00** | 0.00 | 0.02 | **0.11** | 0.04 | 0.25 | **0.19** | 0.07 | 0.28 | **0.13** | 0.11 | 1.33 | **0.12** | 0.11 | 0.17 | **0.11** | 0.10 | 0.12 |
| Pb | **0.51** | 0.25 | 0.76 | **0.64** | 0.37 | 0.93 | **0.54** | 0.28 | 0.83 | **0.60** | 0.40 | 0.77 | **0.53** | 0.46 | 0.75 | **0.39** | 0.10 | 0.85 | **0.51** | 0.23 | 0.88 | **0.44** | 0.10 | 0.66 |
| Se | **0.66** | 0.00 | 0.79 | **0.63** | 0.19 | 2.51 | **0.73** | 0.16 | 0.97 | **0.60** | 0.19 | 1.12 | **0.49** | 0.24 | 0.88 | **0.66** | 0.00 | 3.70 | **2.13** | 1.46 | 2.72 | **1.70** | 1.57 | 2.63 |
| Sr | **86.1** | 31.6 | 125.6 | **198.0** | 94.8 | 265.2 | **195.9** | 138.6 | 223.2 | **153.1** | 81.7 | 203.7 | **86.4** | 55.7 | 112.3 | **52.4** | 32.8 | 91.9 | **49.9** | 36.5 | 78.4 | **51.5** | 29.0 | 97.2 |
| V | **0.08** | 0.00 | 0.16 | **0.03** | 0.00 | 0.09 | **0.03** | 0.00 | 0.09 | **0.05** | 0.00 | 0.14 | **0.01** | 0.00 | 0.17 | **0.06** | 0.00 | 0.20 | **0.08** | 0.00 | 0.25 | **0.17** | 0.02 | 0.30 |
| Zn | **5.54** | 2.83 | 10.98 | **7.90** | 5.14 | 10.49 | **4.84** | 2.93 | 7.81 | **3.21** | 1.21 | 6.63 | **8.92** | 6.13 | 16.86 | **25.20** | 10.10 | 35.07 | **19.20** | 13.34 | 31.81 | **10.24** | 2.09 | 34.16 |

* - x 10^3^

Table ES4. Sources of variability in the concentrations of elements [log(x+1)-transformed] (average percentage dissimilarity) in the eggshells of Great Cormorants and Grey Herons from the studied mixed colonies according to a SIMPER analysis.

| Element | Average dissimilarity | Contribution (%) |
| --- | --- | --- |
| *Inter- species and inter-colony dissimilarity* | | |
| Al | 1.59 | 17.7 |
| Zn | 1.03 | 11.4 |
| Mn | 1.00 | 11.1 |
| Ni | 0.96 | 10.7 |
| Sr | 0.96 | 10.7 |
| Fe | 0.90 | 10.1 |
| *Inter- species dissimilarity* | | |
| Al | 2.29 | 20.9 |
| Ni | 1.53 | 14.0 |
| Mn | 1.20 | 10.9 |

Only elements with a contribution of ≥5% are presented

Table ES5. Results of two-way PERMANOVA analyses for particular elements (with element concentration as analyzed variable and species, site and species x site interaction as predictors).

| Al | df | F | p | As | df | F | p | Ca | df | F | p |
| --- | --- | --- | --- | --- | --- | --- | --- | --- | --- | --- | --- |
| Species | 1 | 258.26 | 0.0001 | Species | 1 | 45.462 | 0.0001 | Species | 1 | 3633.2 | 0.0001 |
| Colony | 7 | 4.2444 | 0.0001 | Colony | 7 | 1.3903 | 0.1400 | Colony | 7 | 27.302 | 0.0001 |
| Interaction | 7 | 4.7979 | 0.0001 | Interaction | 7 | 2.2256 | 0.0029 | Interaction | 7 | 7.4273 | 0.0001 |
| Cd | df | F | p | Cr | df | F | p | Cu | df | F | p |
| Species | 1 | 12.664 | 0.0001 | Species | 1 | 36.661 | 0.0001 | Species | 1 | 451.08 | 0.0001 |
| Colony | 7 | 4.6865 | 0.0001 | Colony | 7 | 7.5745 | 0.0001 | Colony | 7 | 29.337 | 0.0001 |
| Interaction | 7 | 6.1345 | 0.0001 | Interaction | 7 | 7.1246 | 0.0001 | Interaction | 7 | 28.208 | 0.0001 |
| Fe | df | F | p | Hg | df | F | p | Mg | df | F | p |
| Species | 1 | 74.76 | 0.0001 | Species | 1 | 14.653 | 0.0001 | Species | 1 | 326.32 | 0.0001 |
| Colony | 7 | 27.965 | 0.0001 | Colony | 7 | 5.9778 | 0.0001 | Colony | 7 | 9.1078 | 0.0001 |
| Interaction | 7 | 3.8266 | 0.0003 | Interaction | 7 | 2.708 | 0.0006 | Interaction | 7 | 8.2603 | 0.0001 |
| Mn | df | F | p | Mo | df | F | p | Ni | df | F | p |
| Species | 1 | 141.67 | 0.0001 | Species | 1 | 11.889 | 0.0004 | Species | 1 | 695.67 | 0.0001 |
| Colony | 7 | 5.9181 | 0.0001 | Colony | 7 | 3.4353 | 0.0006 | Colony | 7 | 18.089 | 0.0001 |
| Interaction | 7 | 6.7689 | 0.0001 | Interaction | 7 | 4.5325 | 0.0001 | Interaction | 7 | 12.532 | 0.0001 |
| Pb | df | F | p | Se | df | F | p | Sr | df | F | p |
| GAT | 1 | 11.384 | 0.0001 | Species | 1 | 11.703 | 0.0001 | Species | 1 | 52.55 | 0.0001 |
| KOL | 7 | 1.4608 | 0.0972 | Colony | 7 | 7.0611 | 0.0001 | Colony | 7 | 73.374 | 0.0001 |
| Interaction | 7 | 1.7554 | 0.0433 | Interaction | 7 | 4.6894 | 0.0001 | Interaction | 7 | 7.5661 | 0.0001 |
| V | df | F | p | Zn | df | F | p |  |  |  |  |
| GAT | 1 | 9.7493 | 0.0002 | Species | 1 | 1.5712 | 0.2013 |  |  |  |  |
| KOL | 7 | 1.6676 | 0.0641 | Colony | 7 | 63.34 | 0.0001 |  |  |  |  |
| Interaction | 7 | 0.67766 | 0.5465 | Interaction | 7 | 10.216 | 0.0001 |  |  |  |  |


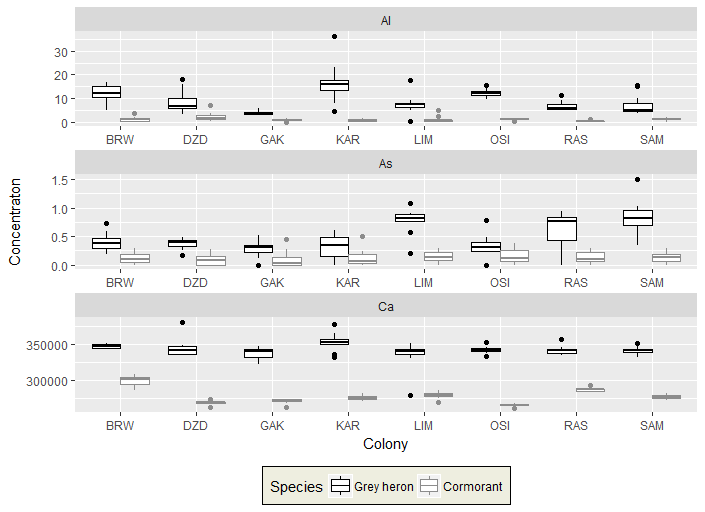


Fig. ES1. Concentrations of Al, As, Ca in post-hatched eggshells of Great Cormorants and Grey Herons breeding in 8 mixed colonies in Poland. Colony codes – see Table 1.


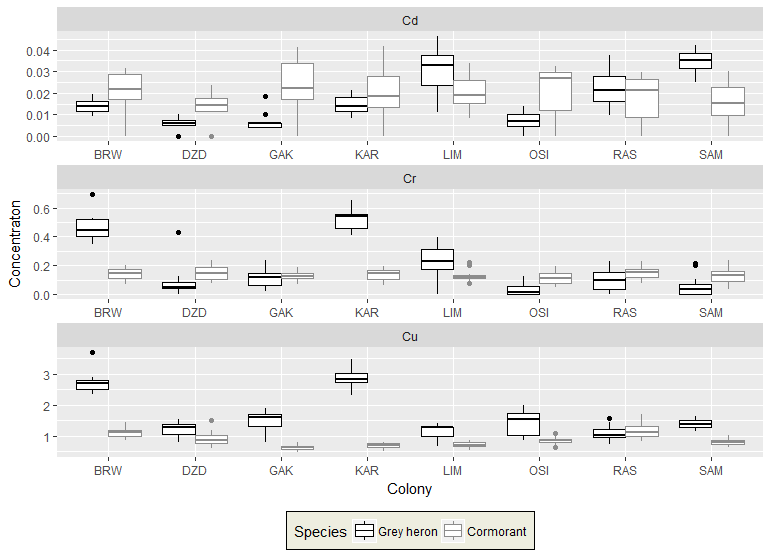


Fig. ES2. Concentrations of Cd, Cr, Cu in post-hatched eggshells of Great Cormorants and Grey Herons breeding in 8 mixed colonies in Poland. Colony codes – see Table 1.


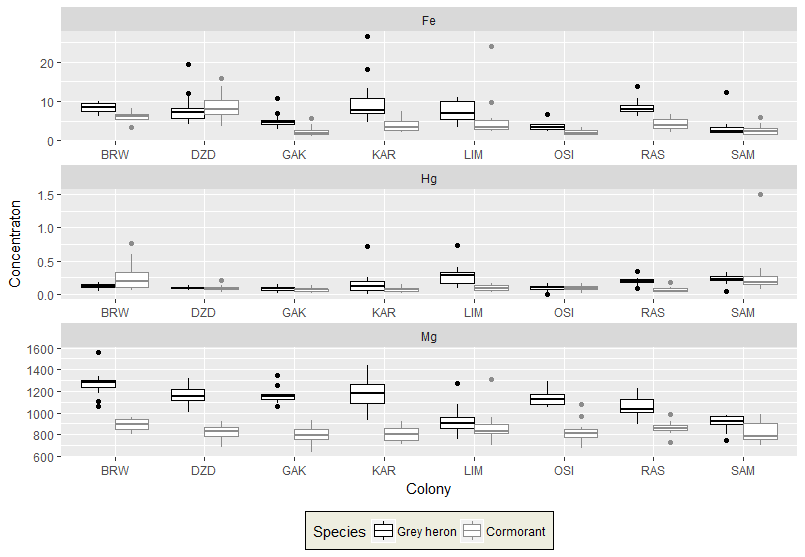


Fig. ES3. Concentrations of Fe, Hg, Mg in post-hatched eggshells of Great Cormorants and Grey Herons breeding in 8 mixed colonies in Poland. Colony codes – see Table 1.


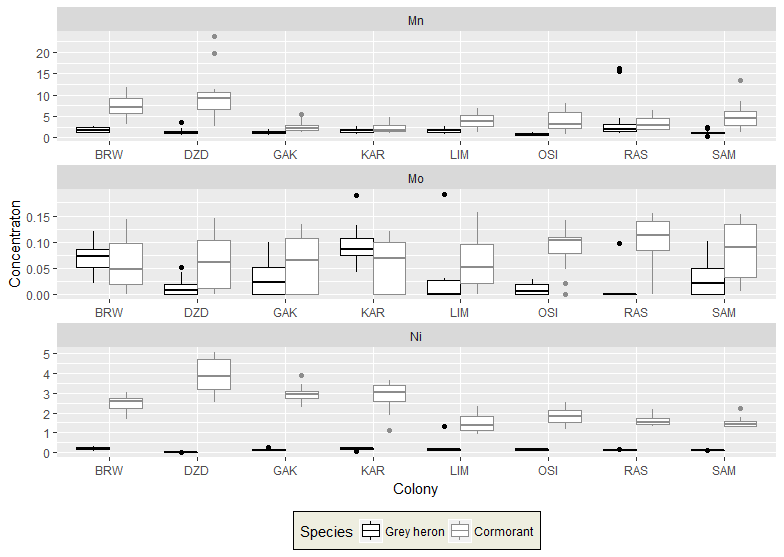


Fig. ES4. Concentrations of Mn, Mo, Ni in post-hatched eggshells of Great Cormorants and Grey Herons breeding in 8 mixed colonies in Poland. Colony codes – see Table 1.


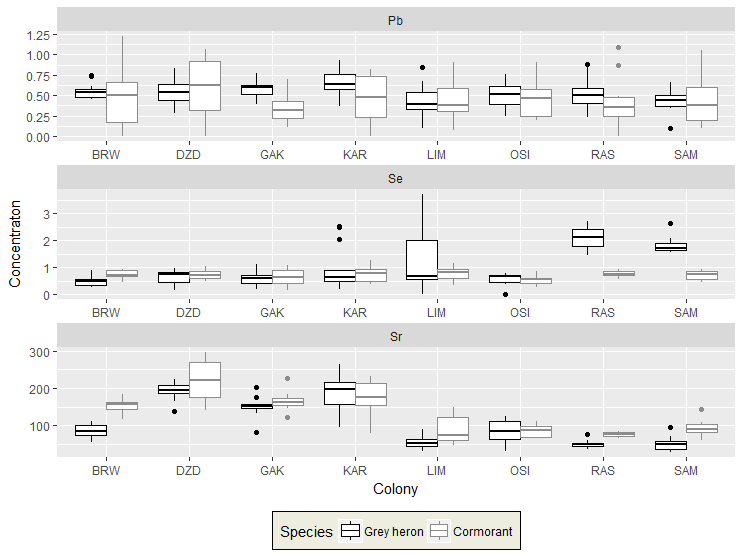


Fig. ES5. Concentrations of Pb, Se, Sr in post-hatched eggshells of Great Cormorants and Grey Herons breeding in 8 mixed colonies in Poland. Colony codes – see Table 1.


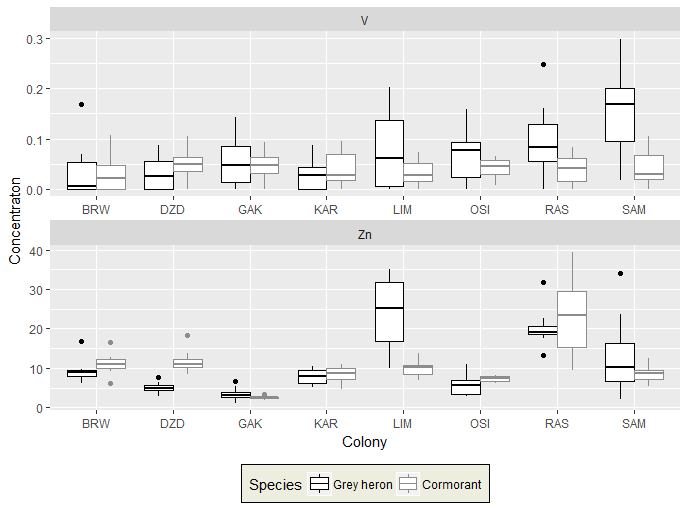


Fig. ES6. Concentrations of V, Zn in post-hatched eggshells of Great Cormorants and Grey Herons breeding in 8 mixed colonies in Poland. Colony codes – see Table 1.
